# The combination of stem cells and tissue engineering: an advanced strategy for blood vessels regeneration and vascular disease treatment

**DOI:** 10.1186/s13287-017-0642-y

**Published:** 2017-09-15

**Authors:** Ying Wang, Pei Yin, Guang-Liang Bian, Hao-Yue Huang, Han Shen, Jun-Jie Yang, Zi-Ying Yang, Zhen-Ya Shen

**Affiliations:** 1 Department of Cardiovascular Surgery & Institute of Cardiovascular Science, First Affiliated Hospital of Soochow University, Suzhou, Jiangsu China; 2 Department of Cardio-Thoracic Surgery, Taixing People’s Hospital, Taixing, Jiangsu China; 3 Department of Cardio-Thoracic Surgery, Jingjiang People’s Hospital, Jingjiang, Jiangsu China

**Keywords:** Stem cells, Vascular tissue engineering, Scaffold material, Regeneration

## Abstract

Over the past years, vascular diseases have continued to threaten human health and increase financial burdens worldwide. Transplantation of allogeneic and autologous blood vessels is the most convenient treatment. However, it could not be applied generally due to the scarcity of donors and the patient’s condition. Developments in tissue engineering are contributing greatly with regard to this urgent need for blood vessels. Tissue engineering-derived blood vessels are promising alternatives for patients with aortic dissection/aneurysm. The aim of this review is to show the importance of advances in biomaterials development for the treatment of vascular disease. We also provide a comprehensive overview of the current status of tissue reconstruction from stem cells and transplantable cellular scaffold constructs, focusing on the combination of stem cells and tissue engineering for blood vessel regeneration and vascular disease treatment.

## Background

Annually, thousands of people die of aortic aneurysm, thoracic aortic dissection, and other vascular diseases. As administered drugs cause a number of chemical reactions in the body and can even lead to autoimmune complications, advances in regenerative medicine and tissue engineering are extremely beneficial. Tissue engineering and regenerative medicine not only offer fast recovery but also lessen the medical and economic burden among patients suffering from vascular diseases. We have accumulated a lot of information on and developed techniques for biofabrication and stem cell biology, but the effectiveness of clinical applications of these techniques remains to be elucidated. This review highlights advances in the regenerative medicine and tissue engineering fields with respect to vascular diseases. In particular, we discuss scaffold-free cell therapies for blood vessel defects as well as the various stem cells used for blood vessel engineering.

## Tissue engineering for vascularized tissues

Vascular diseases are pivotal causes of mortality and morbidity in developed countries. As reviewed by Zaragosa et al. [1], both genetic and environmental factors are associated with cardiac and vascular complications; therefore, these complex multifactorial pathologies are very difficult to prevent [1]. Although prior studies have emphasized that new drugs and innovative devices have improved the quality of life for patients inflicted with vascular diseases, these have not necessarily reduced the mortality and morbidity rate [2]. Transplantation has provided a new therapeutic path as it offers an immediate “cure” by replacing the damaged tissues or organs with normally functional substitutes.

Successful treatment of vascular diseases has been limited, however, due to a lack of suitable autologous tissue to restore injured vessels or to serve as vascular conduits to replace or bypass diseased or occluded vessels. On the other hand, although immunosuppressive agents have reduced the mortality and morbidity associated with organ failure, they increase the risk of infection, cancer, and cardiovascular diseases [2]. Hence, tissue engineering has been proposed as an alternative treatment that might overcome these problems by replacing the damaged tissue or organ function with constructs which are biofabricated based on the required tissue or organ features [2]. In particular, vascular tissue engineering plays an important role in increasing life expectancy and preservation of extremities [3]. In addition, tissue engineering is designed to produce biomimetic constructs resembling normal tissues to replace damaged tissues. Moreover, the main objective of tissue engineering is the restoration of function through the delivery of living elements which become integrated in the patient [4].

## Vascular tissue engineering technology

Tissue engineering strategies have three basic components: firstly, the cells or source which must express the appropriate genes and maintain the appropriate phenotype in order to preserve the specific function of the tissue [5]; secondly, the bioreactive agents or signals that induce cells to function; and thirdly, the scaffolds that house the cells and act as a substitute for the damaged tissue [6]. The source may be either embryonic stem cells (ESC) or adult stem cells (ASC), the scaffolds may be categorized as synthetic, biological, or composite, and the signals may include growth factors/cytokines, adhesion factors, and bioreactors [7].

**Fig. 1:**
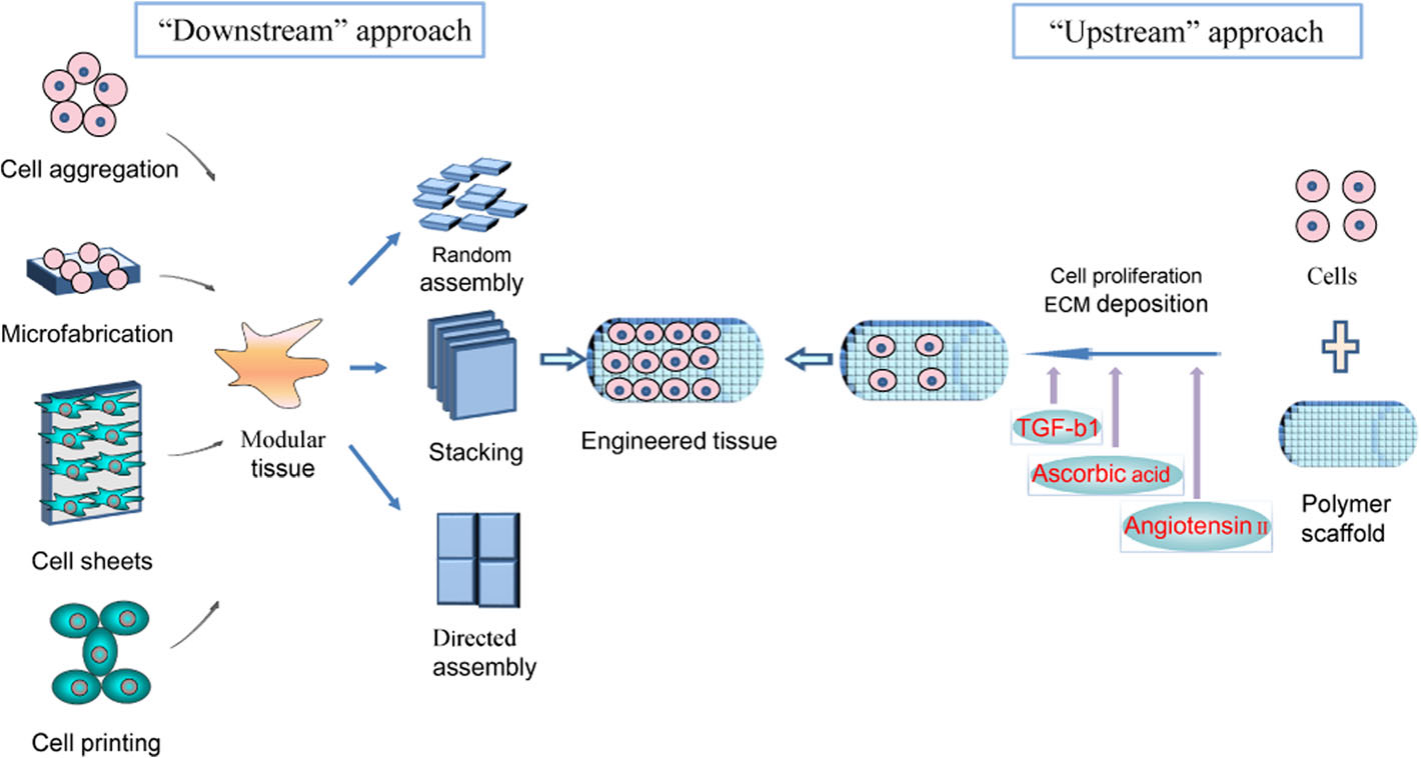
Downstream and upstream approaches to tissue engineering. In the downstream approach, multiple methods are used for creating modular tissues, which are then assembled into engineered tissues with specific micro-architectural features. In the upstream approach, cells and biomaterial scaffolds are combined and cultured until the cells fill the support structure to create an engineered tissue

Currently, downstream and upstream approaches (Fig. 1) in tissue engineering have been continuously investigated by many groups as the most promising tissue engineering approaches. The downstream approach usually employs implantation of precultured cells and synthetic scaffold complexes into the defect area. The cells or source, generally isolated from host target tissues, are expanded in vitro and preseeded into the scaffold to provide a porous three-dimensional structure that accommodates the seeded cells and forms the extracellular matrix (ECM) [8, 9]. Subsequently, multiple methods such as cell aggregation, micro-fabrication, cell sheeting, and cell printing are utilized to generate modular tissues. These are then assembled through random assembly, stacking of cell sheets, or directed assembly into engineered tissues with specific micro-architectural features. Thereafter, the engineered tissue is transplanted into the defective area. This approach allows scientists to finely transform the nanostructure of materials by balancing polymer degradation rates with ECM production and cellular infiltration which results in increased cell binding sequences, enzymatic cleavage sites, and tethering of chemoattractant molecules [10, 11].

Conversely, in the upstream approach, there are two ways to manufacture the engineered tissue: (1) cells and biomaterial scaffolds are combined and cultured until the cells fill the support structure to create an engineered tissue [10]; or (2) acellular scaffolds with incorporated biomolecules are delivered immediately after injury. The biomolecules are released from the scaffolds in a controlled manner, and they may recruit progenitor cells in the injured area and promote their proliferation and differentiation, eventually repairing the injured tissues [8, 9].

## Stem cell sources for vascular tissue engineering

Vascular cells used for tissue engineering are derived from different types of adult stem cells and progenitor cells. Adult stem cells are isolated from different sources, including bone marrow (BM), adipose tissue, hair follicle (HF), umbilical cord (UC), and so on (Table 1).

**Table 1:** In vitro studies of vascular lineage differentiation from multipotent stem cells

Stem cell source	Vascular lineage	Cell origin	Differentiation factors	Scaffold	Selection and characterization	Implant site	Implant/culturing duration	Patency	Reference
BM-MSCs	SMC/EC	Rabbit	FBS; L-glutamine; L-ascorbic-acid-2-phosphate	2-mm diameter mandrel	Suture-holding; strength; platelet counts	CA	4 weeks	100%	[19]
BM-MSCs	SMC/EC	Ovine	FBS; VEGF; bFGF; ascorbic acid; TGF-b1	CA decellularized	SM a-actin; PKH26; vWF	CA	5 months	100%	[20]
AD-SCs	SMC	Human	Media-199; FBS; angiotensin II; TGF-b1; phingosylphosphorylcholine	Decellularized; greater saphenous vein	SMC; calponin; caldesmon; MHC; ANG; APC; TGFb1	Bioreactor system	-	-	[21]
AD-SCs	SMC	Human	TGF-b1; BMP4; FBS; penicillin-streptomycin	PGA	Smooth muscle alpha actin; a-SMA; calponin; SM-MHC;	Bioreactor system	8 weeks	-	[22]
HF-SCs	SMC	Human	FBS; isobutyl-methylxanthine; dexamethasone; insulin; indomethacin	Mandrel of poly(di-methylsiloxane)	bFGF; TGF-b1; a-SMA; calponin	-	2 weeks	-	[23]
HF-SCs	SMC	Newbon lamb	DMEM; FBS	SIS	EGFP; a-SMA; calponin; MHC	-	2 weeks	-	[24]
ESCs	SMC	Nude mice	All-*trans*-retinoid acid (RA); DMSO	3D macro-porous NF scaffolds	α-SM; SM-MHC; OCT4	Subcutaneous pockets on nude mice	2 weeks	-	[25]
hiPSCs	SMC	Human	EB; FBS; NEAA; glutamine; mercaptoethanol	PGA	a-SMA; SM-MHC; calponin; SM22a	Nude rats AA	2 weeks	100%	[26]
HUVECs	HAF-HUVEC	Human	FCS; EGM2	Bioreactor system	Collagen IV; VEGF; a-SMA; Ki67	-	2 weeks	-	[27]
PB-EPC	PB-EPC	Ovine	FBS; medium with low glucose; L-glutamine	Decell porcine CA	Actin; MHC	Arterial interposition	4 months	100%	[28]

*AD* adipose, *bFGF* fibroblast growth factor basic, *BM* bone marrow, *EB* embryoid body, *EC* endothelial cell, *EPC* endothelial progenitor cell, *ESC* embryonic stem cell, *FBS* fetal bovine serum, *HF* hair follicle, *hiPSC* human induced pluripotent stem, *MSC* mesenchymal stem cell, *PB* peripheral blood, *PGA* phosphoglyceric acid, *SC* stem cell, *SIS* small intestinal submucosa, *SMC* smooth muscle cell, *TGF* transforming growth factor, *VEGF* vascular endothelial growth factor, *PGA* polyglycolic acid, *AA* abdominal aorta, *CA* carotid artery, *EGFP* enhanced green fluorescent protein, *NF* nanofibrous, *a-SMA* alpha-smooth muscle antibody, *APC* antigen presenting cell

### Bone marrow mesenchymal stem cells

As early as 1993, Galmiche et al. [12] documented that stromal cells from human long-term marrow cultures are mesenchymal cells that differentiate along a vascular smooth muscle differentiation pathway. Various factors such as soluble growth factors, cell–cell contact, mechanical stimulation, and extracellular matrix substrate proteins are known to have an effect on the differentiation of mesenchymal stem cells (MSCs).

BM-MSCs are the most commonly used MSC type with the source of harvested bone marrow typically being the iliac crest. Tissue engineered vascular grafts created using BM-MSCs have been used in vivo and shown positive results. Zhao et al. [13] obtained ovine BM-MSCs and differentiated them into endothelial cell (ECs) and smooth muscle cells (SMCs) before seeding them onto decellularized scaffolds. EC differentiation was performed by culturing BM-MSCs in DMEM supplemented with fetal bovine serum (FBS), penicillin-streptomycin, glutamine, vascular endothelial growth factor, basic fibroblast growth factor, and ascorbic acid. SMC differentiation was performed by culturing BM-MSCs in DMEM supplemented with FBS, penicillin-streptomycin, insulin, and transforming growth factor-β1 (TGF-β1). When seeded constructs were implanted autologously into the sheep model they remained patent and antithrombotic, were comprised of vascular components, and were mechanically stable for 5 months. Unseeded controls occluded in 2 weeks.

Hjortnaes et al. [14] showed that their dual-seeded MSC and endothelial progenitor cell (EPC) phosphoglyceric acid (PGA)/poly-L-lactic acid (PLLA) graft showed strong proteolytic activity, patency, and the presence of seeded cells in vivo by injecting near-infrared agents and labeling their BM-MSCs with green fluorescent protein (GFP). They noted that GFP expression decreased over time, further supporting that the grafts are repopulated over time with host cells, which is a trend also seen with undifferentiated BM mononuclear cells. They also stained explanted samples to show expression of Mac-3, a macrophage marker, as well as matrix metalloproteinases (MMPs) to indicate an inflammatory-driven response. Hashi et al. [15] showed that BM-MSCs have antithrombotic properties that are similar to those of ECs, and this is in part due to heparin expression on the surface of BM-MSCs. They went on to show that BM-MSCs seeded in biodegradable scaffolds only remain in the construct for 1 week. Despite this, BM-MSCs may only need to be present to provide short-term antithrombogenicity since endothelialization happens between 1 week and 1 month after transplantation and neointimal thickening of acellular grafts stabilizes after the first week. To aid this antithrombogenic nature of BM-MSCs, they have also been genetically modified to express endothelial nitric oxide synthase at levels consistent with native blood vessels [16].

### Adipose-derived stem cells

Adipose-derived stem cells (AD-SCs) are first isolated from the stromo-vascular fraction (SVF) of adipose tissue harvested during liposuction [17]. A few hundred milliliters to a few liters of adipose tissue is routinely liposuctioned from patients, and the lipoaspirate is subsequently digested with collagenase, washed, and filtered. AD-SCs are derived from the filtrate, also known as processed lipoaspirate, after culture for several days in the presence of FBS [17].

AD-SCs have a fibroblastic morphology and MSC-like immunophenotype (CD34+/CD105+/CD45−/CD31− or CD29+/CD44+/CD71+/CD90+/CD105+/STRO-1+/CD49d+/CD31−/CD34−/CD45−) and exhibit a clonal differentiation potential along all mesenchymal lineages [18]. In addition, several studies demonstrated that the differentiation potential of AD-SCs extends beyond mesodermal boundaries and includes neurons [19] and pancreatic islets [20]. Finally, AD-SCs were found to suppress the mixed lymphocyte reaction and lymphocyte proliferation, supporting their potential for allogenic transplantations [21].

More recently, SMCs have been derived from AD-SCs by treatment with TGF-β1 and bone morphogenetic protein 4 (BMP4) and were used on a PGA scaffold to generate the vascular wall [22]. Interestingly, the application of pulsatile force significantly improved both collagen synthesis and vessel wall mechanics [22]; however, the implantability of these vascular grafts was not demonstrated.

Several groups have reported that AD-SCs are a rich source of ECs [23]. In addition to the total AD-SC population, the CD34+/CD31− cell fraction from the gluteal, abdominal, and visceral adipose tissue of human donors expressed endothelial markers and showed enhanced vascularization after implantation in a hind limb ischemia mouse model [24]. Interestingly, the EC differentiation capacity of AD-SCs was not affected by either donor age or vascular disease [25]. However, others showed that, in AD-SC cultures, the CD31 and CD144 endothelial-specific promoters were methylated even after stimulation with EC-promoting growth factors, suggesting that the differentiation potential of AD-SCs toward the endothelial fate might be epigenetically limited [26]. Given the heterogeneity of the SVF, it is not clear whether AD-SC-derived ECs either differentiate directly from a common multipotent progenitor or represent a population of EC progenitors in SVF. Nevertheless, taken together, these studies support the notion that AD-SCs constitute an important stem cell source for vascular regeneration.

### HF-derived stem cells

The HF is a dynamic mini-organ containing cells of ectodermal and mesodermal origin. Anatomically, the HF can be divided into four regions of ectodermal origin: (1) infundibulum; (2) isthmus; (3) suprabulbar region; and (4) bulb. Throughout life, HFs undergo many cycles of growth (anagen), regression (catagen), and quiescence (telogen), suggesting the presence of stem cells that support the process of continuous regeneration. However, the anatomic location where stem cells reside remained elusive until the early 1990s, when experiments were designed to follow the fate of label-retaining cells, showing that these cells resided in the bulge region of the isthmus [27]. This finding was later verified with sophisticated cell-fate mapping experiments [28] that established the bulge as the stem cell niche of the HF. The multipotency of HF-derived stem cells (HF-SCs) has also been demonstrated by coaxing them to differentiate into corneal epithelial cells [29], neurons [30], and myelinating glial cells [31].

The HF is surrounded by the dermal sheath (DS), which contains progenitor cells that maintain and regenerate the dermal papilla (DP), a mesodermal structure underneath the bulb region, which is very important for promoting hair regeneration [32]. The DP and DS of rat HFs were shown to contain MSCs (termed HF-MSCs), which showed similar proliferation and differentiation potential as rat BM-MSCs [33]. Notably, HF-MSCs showed hematopoietic differentiation potential, as displayed by transplantation experiments in which cultured DP cells repopulated the BM of lethally irradiated mice [34]. In their group, we showed that the immunophenotypes of human HF-MSCs are similar to those of BM-MSCs (CD73+/CD105+/CD44+/CD49b+/CD90+/CD309−/CD144−/CD34−/CD45−) and that they are clonally multipotent, as individual cells can be coaxed to differentiate into fat, bone, cartilage, and muscle cells [35]. We also noted that HF-MSCs were less susceptible to culture senescence than BM-MSCs, as they could undergo 45 population doublings and maintained their myogenic differentiation potential, as exhibited by the development of contractile properties even at late passages. When combined with the natural scaffold, small intestinal submucosa (SIS), HF-MSC-derived SMCs generated vascular media with significant vascular contractility and mechanical properties approaching those of native arteries [36], demonstrating the potential of HF-MSCs for use in vascular tissue engineering. Indeed, cylindrical Tissue-engineered vessels (TEV) generated with this approach in our laboratory are currently implanted as interpositional grafts in the arterial circulation of an ovine animal model (unpublished data).

Researchers recently reported a new source of SMCs derived from HF-MSCs. HF-SMCs demonstrated high proliferation and clonogenic potential as well as contractile function [36]. In their study, they aimed at engineering the vascular media using HF-SMCs and a natural biomaterial, namely SIS. Engineering functional vascular constructs required application of mechanical force, resulting in actin reorganization and cellular alignment. In turn, cell alignment was necessary for development of receptor- and nonreceptor-mediated contractility as soon as 24 h after cell seeding. Within 2 weeks in culture, the cells migrated into SIS and secreted collagen and elastin, the two major extracellular matrix components of the vessel wall. At 2 weeks, vascular reactivity increased significantly up to three- to fivefold and mechanical properties were similar to those of native ovine arteries. Taken together, the data demonstrate that the combination of HF-SMCs with SIS resulted in mechanically strong, biologically functional vascular media with potential for arterial implantation.

### Embryonic stem cells

Embryonic stem cells (ESCs) are cells that are derived from preimplantation embryos at the blastocyst stage. To date, researchers have successfully derived ECs and SMCs from human ESC (hESC) lines. Several different strategies have been explored to derive SMCs from hESCs. Progenitor cells such as CD34+ cells have been isolated either from spontaneously differentiated embryoid bodies (EBs) [37] or from co-cultures and then further induced with factors such as TGF-β1, PDGF-BB (the two B subunits of platelet-derived growth factor), retinoic acid, or a combination thereof to aid the differentiation into SMC-like cells [38]. Others have utilized a combination of inductive cell culture medium along with extracellular matrix such as collagen IV or Matrigel [39] to favor SMC differentiation. ECs have also been derived from hESCs by using PECAM antibodies to select for these cells or by isolating a CD34+ progenitor population from 10- to 15-day EBs that is then differentiated into mature ECs and SMCs [37]. Several groups have derived ECs from hESCs using 2D and 3D cultures [40] and demonstrated the ability of these cells to form implantable blood vessels as well as vascularized skeletal tissue [41]. Studies have demonstrated the functionality of ESC-derived cells in animal models. ESC-derived ECs are able to form tube-like structures on Matrigel and formed microvessels [42] when they were transplanted into SCID mice. Using a scalable 2D differentiation system, Wang et al. [42] derived ECs from ESCs which, when transplanted into SCID mice, formed blood conduits that were functional for 150 days. However, the functionality of such ESC-derived cells in engineering human vascular grafts still remains to be tested.

### Induced pluripotent stem cells

The creation of iPSCs is one of the most important biomedical discoveries of our time. Similar to hESCs, iPSCs have been shown to differentiate into a variety of cell types. To date, ECs and EPCs as well as mesenchymal cells have been successfully derived from human iPSCs. The proliferative capacity as well as the functionality of vascular cells derived from iPSCs seem to depend on the differentiation protocol as well as the origin of the iPSCs. However, others studies, such as Lian et al. [43], have been successful in generating mesenchymal cells with high proliferative capacity from human iPSCs. These iPSC-MSCs generated by sorting for CD24−/CD105+ cells had an excellent proliferative capacity with population doublings (PDs) greater than 120 and were able to differentiate into adipocyte, chondrocyte, and osteocyte lineages. These iPS-MSCs had an almost tenfold higher activity of telomerase compared to BM-MSCs. When tested in a mouse model, iPSC-MSCs displayed better functionality in attenuating hind-limb ischemia compared to BM-MSCs. While SMCs have been generated from mouse iPSCs, efforts to derive functional SMCs from human iPSCs are still in the early stages. So far, in a proof-of-concept study, Lee and coworkers [44] demonstrated the ability to derive iPSCs from human vascular cells and differentiated these iPSCs back into SMCs. The derived SMCs were found to have functional characteristics very similar to those of the original parental SMCs from which the iPSCs were created.

### Endothelial precursor cells

EPCs are progenitor cells capable of differentiating into endothelial cells. These cells are particularly useful due to their more desirable harvest location than ECs and can be used to coat vascular grafts in their place. There has been debate over the exact phenotype of an EPC since it has no exclusive markers and the markers for hematopoietic stem cells, EPCs, and ECs overlap [45]. EPCs can be harvested from BM, peripheral blood, and UC blood, but these populations are not completely identical. ECs obtained from cord blood EPCs (CB-EPCs) have shown increased proliferation potential as well as adhesion over peripheral blood (PB-EPCs) or aortic endothelial cells [46]. Also CB-EPCs have been shown to have higher expression of CXCR4, a chemokine receptor implicated in homing capacity, than BM-EPCs. CB-EPCs express less stromal markers (CD105, CD73) than BM-EPCs, but both express similar endothelial markers. During vasculogenesis, CB-EPCs were able to form stable vascular networks whereas BM-EPCs could not [47]. Seven-fold more EPCs are found in CB than PB but PB-EPCs can be mobilized from the BM into circulation using various growth factors, cytokines, drugs, and hormones.

Similar to ECs, CB-EPCs adhere to SMCs and fibronectin and remain adherent when exposed to supraphysiologic stresses, elongate and orient in the direction of flow, and express similar antithrombogenic genes [48]. Dual seeding with EPCs and MSCs was reported by Hjortnaes et al. [14] and Neff et al. [49] and Zhu et al. [50] co-seeded grafts with EPCs and SMCs. By co-seeding EPCs with SMCs, Neff et al. showed an increased medial cellularity, greater contractile responses, and higher expression of both a-actin and myosin heavy chain than EPC-only seeded grafts [49]. Zhu et al. genetically modified EPCs with the A20 gene, a gene linked to suppressing atherogenesis, and coseeded them with SMCs onto decellularized scaffolds [50]. These grafts showed 100% 6-month patency in the carotid artery of rats compared to 0% patency for non-transfected controls.

## Conclusions and future directions

At present, although the previously established treatments for vascular disorders, such as transplantation, surgical reconstruction, use of mechanical and synthetic devices, or administration of metabolic products, are effective, they still have several constraints and complications. Hence, the development of in vitro and in vivo biomimetic constructs for specific target organs or tissues are more suitable for regeneration of damaged vessels.

Throughout recent years many advances have been made toward using adult stem cells clinically, and this includes the development of tissue engineered vascular grafts. By incorporating various progenitor cells such as bone marrow-derived mononuclear cells, mesenchymal stem cells, or endothelial precursor cells into biodegradable materials, vascular grafts can be created that address limitations currently seen with other treatment approaches. Some of these cells have also shown the ability to initiate regenerative processes within the graft to develop tissue mimicking native arteries.

Another intriguing trend is using 3D bio-printing technology to create a vascular channel and perfuse open lumen with cells and matrix. This approach is very promising because the vascular channel is simultaneously printed with cells and matrix in desired 3D patterns. Using different stem cells, or some gene-modified stem cells, various functions will be conferred on the 3D-printed vascularized tissues, such as what growth factors they secrete and the nature of the inflammatory microenvironment. Vascularized tissue fabrication is still a nascent field, but it is an exciting one that holds promise for the creation of clinically viable vascular grafts.

## Abbreviations

AD-SC: Adipose-derived stem cell
BM: Bone marrow
CB: Cord blood
DP: Dermal papilla
DS: Dermal sheath
EB: Embryoid body
EC: Endothelial cell
EPC: Endothelial progenitor cell
ESC: Embryonic stem cell
FBS: Fetal bovine serum
GFP: Green fluorescent protein
hESC: Human embryonic stem cell
HF: Hair follicle
MSC: Mesenchymal stem cell
PB: Peripheral blood
PGA: Phosphoglyceric acid
SIS: Small intestinal submucosa
SMC: Smooth muscle cell
SVF: Stromo-vascular fraction
UC: Umbilical cord.
